# Milk in Boston

**Published:** 1899-08

**Authors:** 


To secure pure-milk delivery in Boston, the Board of Health has had a census of New England cows taken; the cow-stables and their conditions examined by competent inspectors, and adopted a resolution to prohibit the delivery of any milk in Boston except by license of the board. Thus far (June 1) licenses have been issued to three hundred and ninety-one milk-peddlers, who have complied with all the requirements, while a number have been withheld pending changes in milk-rooms, such as trapping ice-chests, building tight partitions, etc. Permits have been isssued to the owners of 1473 shops. Before a permit is granted, the premises are examined to determine that the sanitary conditions are good. A large number of applications have been rejected, and the unsuccessful applicants have been notified not to continue to sell. The rejections have been caused by absence of ice-chests, use of shops as sleeping rooms, presence of water-closets, and, in not a few instances, particularly in the north and west ends, general conditions of filth.

All milk produced in the city of Boston for sale is required to be strained, cooled, or stored as soon as it is drawn from the cow. The board also forbids milk-dealers using in any way a milk-vessel for any other substance than milk, and any person violating this regulation will be liable to forfeiture of license. Every person engaged in the production, storage, transportation, sale, delivery, or distribution of milk, immediately on the occurrence of any case or cases of infectious disease in his family, or among his employés, or within the building or premises where milk is stored, sold or distributed, is required to notify the Board of Health, and at the same time suspend the sale and distribution of milk until authorized to resume the same by the Board of Health.—*Sanitarian*, July, 1899.

